# Capacitive technologies for highly controlled and personalized electrical stimulation by implantable biomedical systems

**DOI:** 10.1038/s41598-019-41540-3

**Published:** 2019-03-21

**Authors:** Marco P. Soares dos Santos, J. Coutinho, Ana Marote, Bárbara Sousa, A. Ramos, Jorge A. F. Ferreira, Rodrigo Bernardo, André Rodrigues, A. Torres Marques, Odete A. B. da Cruz e Silva, Edward P. Furlani, José A. O. Simões, Sandra I. Vieira

**Affiliations:** 1 Centre for Mechanical Technology & Automation (TEMA), University of Aveiro, Aveiro, Portugal; 2 Department of Mechanical Engineering, University of Aveiro, Aveiro, Portugal; 3 Associated Laboratory for Energy, Transports and Aeronautics (LAETA), Porto, Portugal; 4 Institute of Biomedicine (iBiMED), Department of Medical Sciences, University of Aveiro, Aveiro, Portugal; 5 Mechanical Engineering Department, University of Porto, 4200-465 Porto, Portugal; 6 Department of Chemical and Biological Engineering, Department of Electrical Engineering, University at Buffalo, SUNY, Buffalo, NY USA

## Abstract

Cosurface electrode architectures are able to deliver personalized electric stimuli to target tissues. As such, this technology holds potential for a variety of innovative biomedical devices. However, to date, no detailed analyses have been conducted to evaluate the impact of stimulator architecture and geometry on stimuli features. This work characterizes, for the first time, the electric stimuli delivered to bone cellular tissues during *in vitro* experiments, when using three capacitive architectures: stripped, interdigitated and circular patterns. Computational models are presented that predict the influence of cell confluence, cosurface architecture, electrodes geometry, gap size between electrodes and power excitation on the stimuli delivered to cellular layers. The results demonstrate that these stimulators are able to deliver osteoconductive stimuli. Significant differences in stimuli distributions were observed for different stimulator designs and different external excitations. The thickness specification was found to be of utmost importance. *In vitro* experiments using an osteoblastic cell line highlight that cosurface stimulation at a low frequency can enhance osteoconductive responses, with some electrode-specific differences being found. A major feature of this type of work is that it enables future detailed analyses of stimuli distribution throughout more complex biological structures, such as tissues and organs, towards sophisticated biodevice personalization.

## Introduction

The widespread bioapplications of electromagnetic stimulation (E-Stim), for both research and clinical practice, emphasize the versatility of this biophysical method for a wide range of customized therapies. Electric and/or related magnetic stimulation have been used for therapies in fields that include orthopedics, neurology, ophthalmology and psychiatry, among others^1–10^. Positive effects of E-Stim therapies performed by direct current coupling, inductive coupling and capacitive coupling devices have been reported, as well as the influence of stimuli parameters (frequency, field intensity, etc.) on physiological outcomes^11–15^. However, the field needs to advance and be able to deliver highly individualized stimuli required by current and future personalized medicine^12,16–18^. Technology innovation has triggered the development of the cosurface capacitive electrode architecture for highly controlled and personalized electric stimulation systems^1,12,14^. This capacitive technology breaks the current barriers on stimulation capability as it is able to (i) deliver time- and region-dependent stimuli, (ii) generate a great amount of different stimuli, by varying waveform, magnitude, frequency, periodicity, stimulation exposure, etc., and (iii) deliver non-cytotoxic and non-genotoxic stimuli^12^. Importantly, it only requires a substrate and electrodes in the same surface plane, separated from each other by gaps, to carry out such ability. Using this non-complex design, the number and shape of electrodes can be personalized, even for high demanding applications requiring very large-scale microelectrode arrays, as found in transcranial electric stimulation devices and electrostimulative bone implants^12,16^. Each electrode can be independently controlled according to personalized excitations to provide target-oriented stimulations^12,16^. The potential of cosurface electric technology was already highlighted by *in vitro* and *in vivo* analyses, emphasized for intracorporeal and extracorporeal therapies, principal and coadjuvant therapies, and recognized for both invasive and non-invasive therapies^1,12,14^. Despite its significant impact, the analysis of stimuli distribution and dynamics on physiological tissues is lacking, although it is of upmost importance in the design of bioelectric medical devices^12,18^. Nevertheless, a detailed understanding of the electric field behavior is essential for the development of personalized E-Stim therapies^12,18^. This paper provides, for the first time, computational models of striped, interdigitated and circular cosurface capacitive stimulators to numerically simulate the stimuli delivered to bone cells in culture during the principal stages of osteoblast differentiation, a key event during bone remodeling. Breakthroughs achieved in this study include a unique insight into the influence of design and voltage excitation on electric field dynamics, as well as the validation of simplified models. The ultimate goal of this study is to support the design of innovative active bioelectric medical devices based on biophysical stimulation, and their clinical translation.

## Cosurface Capacitive Architectures

**Figure 1:**
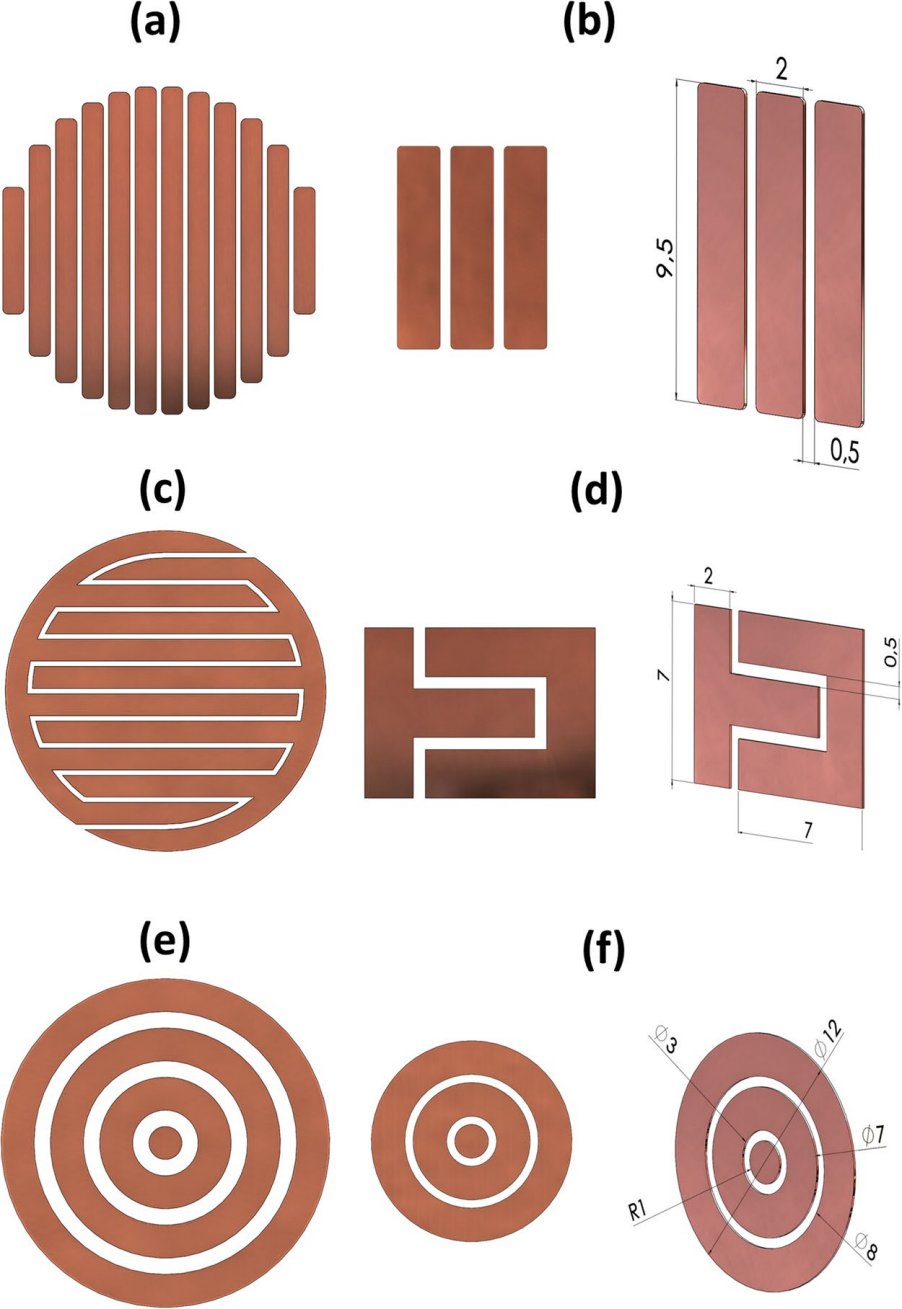
Cosurface capacitive architectures analysed in this study: (**a**) M-pattern of the stripped pattern; (**b**) S-pattern of the stripped pattern; (**c**) M-pattern of the interdigitated pattern; (**d**) S-pattern of the interdigitated pattern; (**e**) M-pattern of the circular pattern; (**f**) S-pattern of the circular pattern.
- **Interdigitated pattern**: the M-pattern is only composed by 2 electrodes, each one with 6 re-entrant stripes of different length (according to the circular border of culture dishes) and 0.5 mm apart, as illustrated by Fig. 1c. The S-pattern is also a patterned zoom-in performed around the mid-point of the M-pattern (up to the stripes’ borders), resulting in electrodes of different geometry and 9.5 mm of overall length (Fig. 1d).
- **Circular pattern**: the M-pattern was modeled with 7 horizontally arranged electrodes (Fig. 1e), 0.5 mm apart from each other; and the S-pattern with 3 electrodes, corresponding to the smaller diameter electrodes of the M-pattern (Fig. 1f).

The electrode stimulation architectures considered in this study were designed for the delivery of controllable electric fields to bone cells cultured on plastic dishes^12^. All electrode patterns were modeled as electrode stripes with 2 mm wide and 1 mm thickness, and the influence of these geometric variables was explored. These patterns were designed to cover almost the entire area of the bottom surface of the culture dishes, such that the entire cell culture can be stimulated. Macro-patterns (M-patterns) were developed for setups using cell culture dishes of 35 mm in diameter, while simplified patterns (S-patterns) were here used and validated to reduce the computational costs required for simulation purposes:

- **Stripped pattern**: the M-pattern comprises 12 electrodes horizontally arranged with different lengths, as shown in Fig. 1a. A 0.5 mm gap between electrodes was parameterized, but the effects of its variation were also observed. The S-pattern (Fig. 1b) is only composed by 3 coplanar electrodes but with equal length (9.5 mm). Similar width and thickness to the ones of the M-pattern were used.

**Figure 2:**
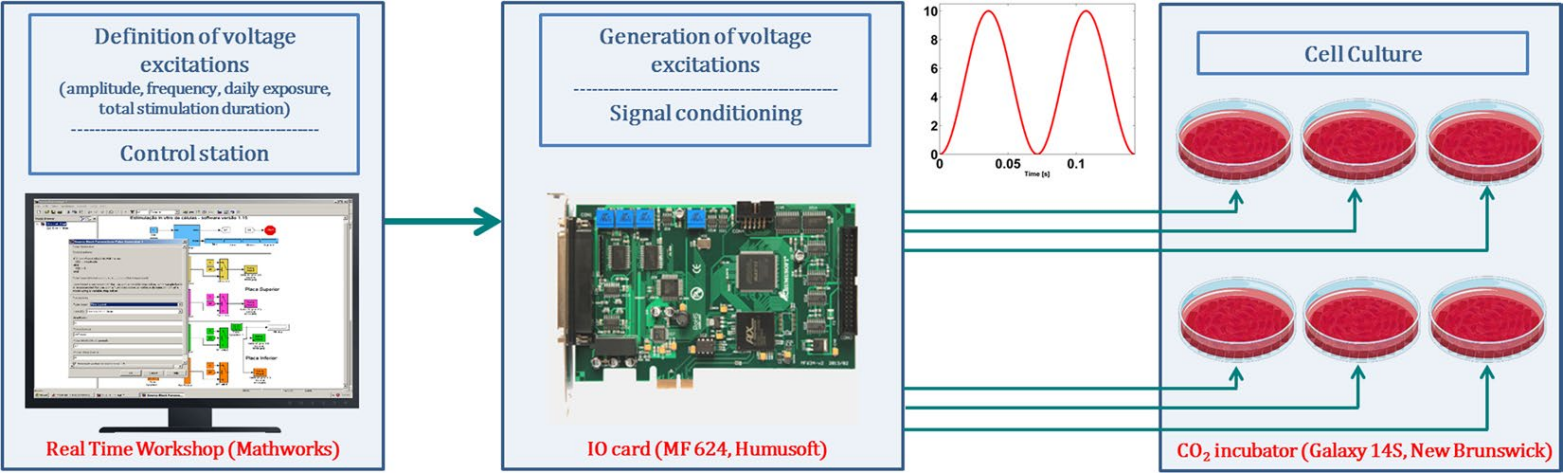
Schematics of the overall stimulation system, which is effective for electric stimulation using stripped, interdigitated and circular patterns. It also refer details of the experimental apparatus implemented to carry out the *in vitro* tests.

A parallel architecture was also modeled for comparative purposes (used as control). Its M-pattern was designed with 32 mm diameter electrodes, 1.5 mm apart from each other. This pattern only differs from the S-pattern due to a lower diameter of its electrodes (12 mm). The overall schematics of the stimulation system (effective for all patterns analysed in this study) is illustrated in Fig. 2. It includes a control station, generation of excitations (powering the electrodes) and cell culturing.

## Computational Models

**Figure 3:**
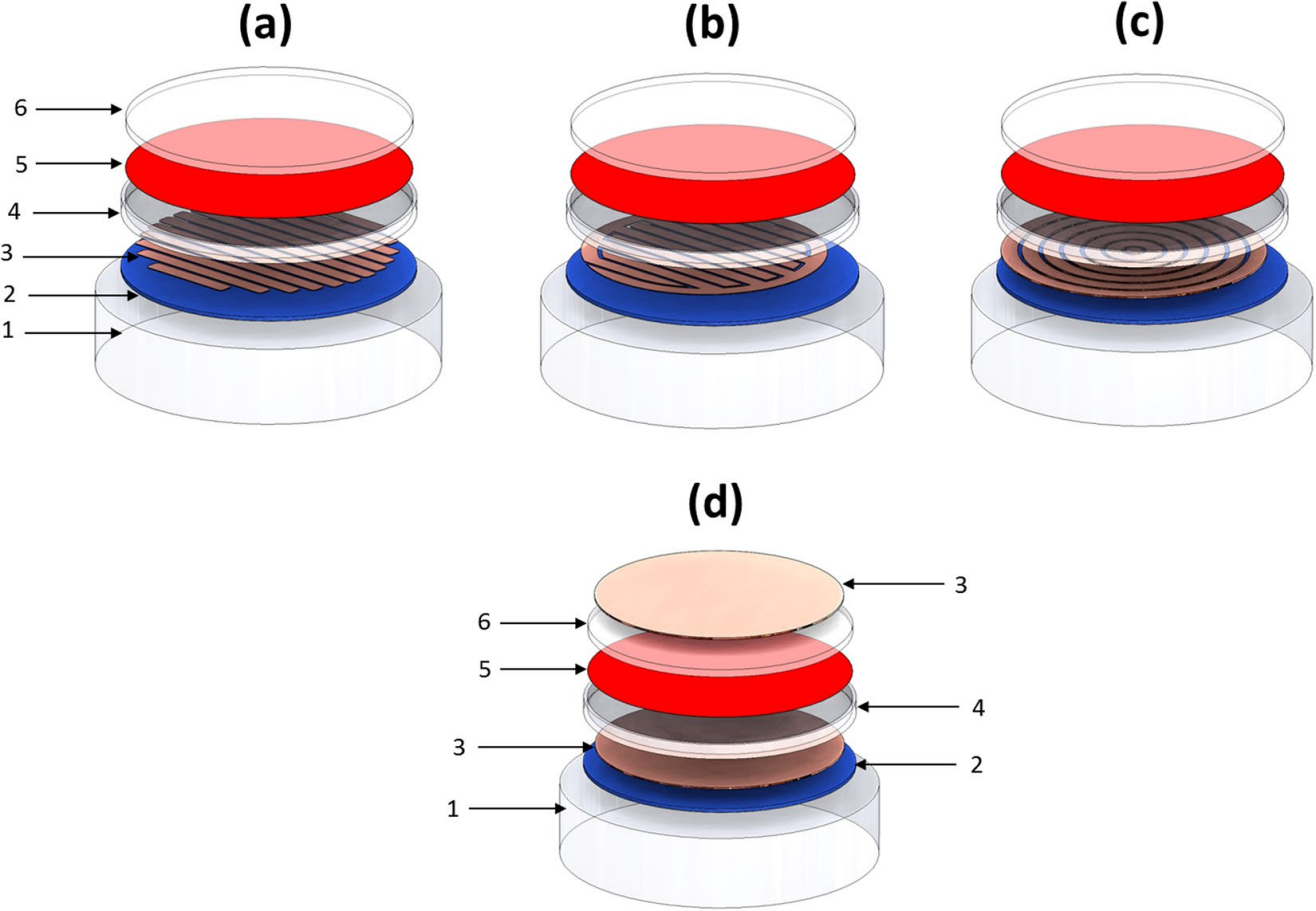
M-models of cosurface capacitive architectures (exploded view): (**a**) Striped pattern; (**b**) Interdigitated pattern; (**c**) Circular pattern. M-models of the parallel architecture (**d**). Domains: 1 - Air; 2 - Polymeric substrate; 3 - Electrodes (M-pattern or S-pattern); 4 - Culture dish; 5 - Cellular layer (proliferation stage) or cellular tissue (differentiation stage); 6 - Culture medium (a liquid solution). S-models are similar to M-models: only domain 3 is essentially different, differing the use of the S-patterns instead of M-patterns.
- A M-model and a S-model for the M-patterned and S-patterned interdigitated stimulator, respectively (Fig. 3b);
- A M-model and a S-model for the M-patterned and S-patterned circular stimulator, respectively (Fig. 3c); and
- A M-model and a S-model for the parallel stimulator (Fig. 3d).

The electric field stimuli provided by our cosurface capacitive stimulation systems was simulated using 8 finite element computational models:

- A macro-scale model (M-model) and a simplified model (S-model) for the M-patterned and S-patterned striped stimulator, respectively (Fig. 3a);

Both M-models and S-models were designed considering apparatuses recently validated in *in silico* and *in vitro* to analyze the effects of electromagnetic stimulation throughout proliferation and differentiation stages of osteoblastic MC3T3 cells^12,19,20^. Each model was built using 6 domains: culture medium (a liquid solution), cellular layer (less dense, as it exists throughout the cells proliferation and differentiation stage) or cellular tissue (when at the end of the differentiation stage), polymeric culture dish, electrodes (M-pattern or S-pattern), polymeric substrate and air. Each pattern of copper electrodes was positioned over a polycarbonate substrate and under a polystyrene culture dish, which contains a cell culture composed of a cellular or a tissue layer (depending on the stage) and a culture medium (Fig. 3a–c). Proliferation and differentiation stages were considered approximately homogeneous phases^12^. As MC3T3 cells are highly adherent to polystyrene surfaces throughout cell culture tests, the cellular or tissue layer was positioned above the culture dish and covered by the liquid solution^12,21^. The fully differentiation stage differs from the proliferation stage by the double thickness of the adherent organic layer, now resembling an organized cellular tissue mainly composed by MC3T3 cells and type-I collagen (a protein that corresponds to approximately 90% of the bone organic matrix)^12^. Table 1 presents the dimensions of each domain for both the M- and S-models, whereas the electric and magnetic properties of their organic and inorganic materials are described in Table 2. These apparatuses ensure no cell-electrode contact, providing an effective therapeutic methodology that can be used in innovative orthopedic medicine, even though models can be easily rebuilt for other medical applications^12,22–26^. Polymeric dishes and substrates were used as they exhibit very high electrical resistivity. A material evidencing very high electrical conductivity properties was considered for all patterned electrodes.

**Table 1:** Dimensions of each domain for both M- and S-models of striped, interdigitated and circular cosurface capacitive stimulators.

Domain	Dimensions of domains							
	M-model				S-model			
	Striped	Interdigitated	Circular	Parallel	Striped	Interdigitated	Circular	Parallel
Culture medium (liquid solution)	34 mm diam^a^; 1 mm thick				13 mm diam; 1 mm thick			
Cellular layer (proliferation)	34 mm diam; 10 *μ*m thick				13 mm diam; 10 *μ*m thick			
Cellular tissue (differentiation)	34 mm diam; 20 *μ*m thick				13 mm diam; 20 *μ*m thick			
Culture dish	35 mm diam; 0.5 mm thick; 2 mm height				14 mm diam; 0.5 mm thick; 2 mm height			
Electrodes (M- and S-pattern)	12–32 mm length; 2 mm wide; 1 mm thick	32 mm diam; 2 mm wide; 1 mm thick	2–32 mm diam; 2 mm wide; 1 mm thick	34 mm diam; 1 mm thick	9.5 mm length^b^; 2 mm wide; 1 mm thick	9.5 mm length^c^; 2 mm wide; 1 mm thick	2–12 mm diam; 2 mm wide; 1 mm thick	13 mm diam; 1 mm thick
Substrate	35 mm wide; 0.5 mm thick				14 mm wide; 0.5 mm thick			
Air^d^	41 mm diam; 9.5 mm height				20 mm diam; 9.5 mm height			

^a^Diameter.

^b^Each stripe.

^c^Overall length.

^d^Dimensions of the ‘Air’ domain were obtained by convergence analysis (section ‘Methods’).

**Table 2:** Electric and magnetic properties of their organic and inorganic materials composing the striped, interdigitated and circular cosurface capacitive stimulators.

Domain	Relative electric permittivity	Electric conductivity [S/m]	Relative magnetic permeability	Reference
Culture medium (liquid solution)	73	1.6	1	^50, 51^
Cellular layer (proliferation)	73	1.2 × 10^−7^	1	^50, 52^
Cellular tissue (differentiation)	73	1.2 × 10^−7^	1	^53, 54^
Culture dish	2.6	6.7 × 10^−14^	1	^12, 55^
Electrodes (M- and S-pattern)	1	6 × 10^7^	1	^12^
Substrate	3	6.7 × 10^−14^	0.866	^55^
Air	1	0	1	^12^

## Excitations Powering the Stimulators

**Figure 4:**
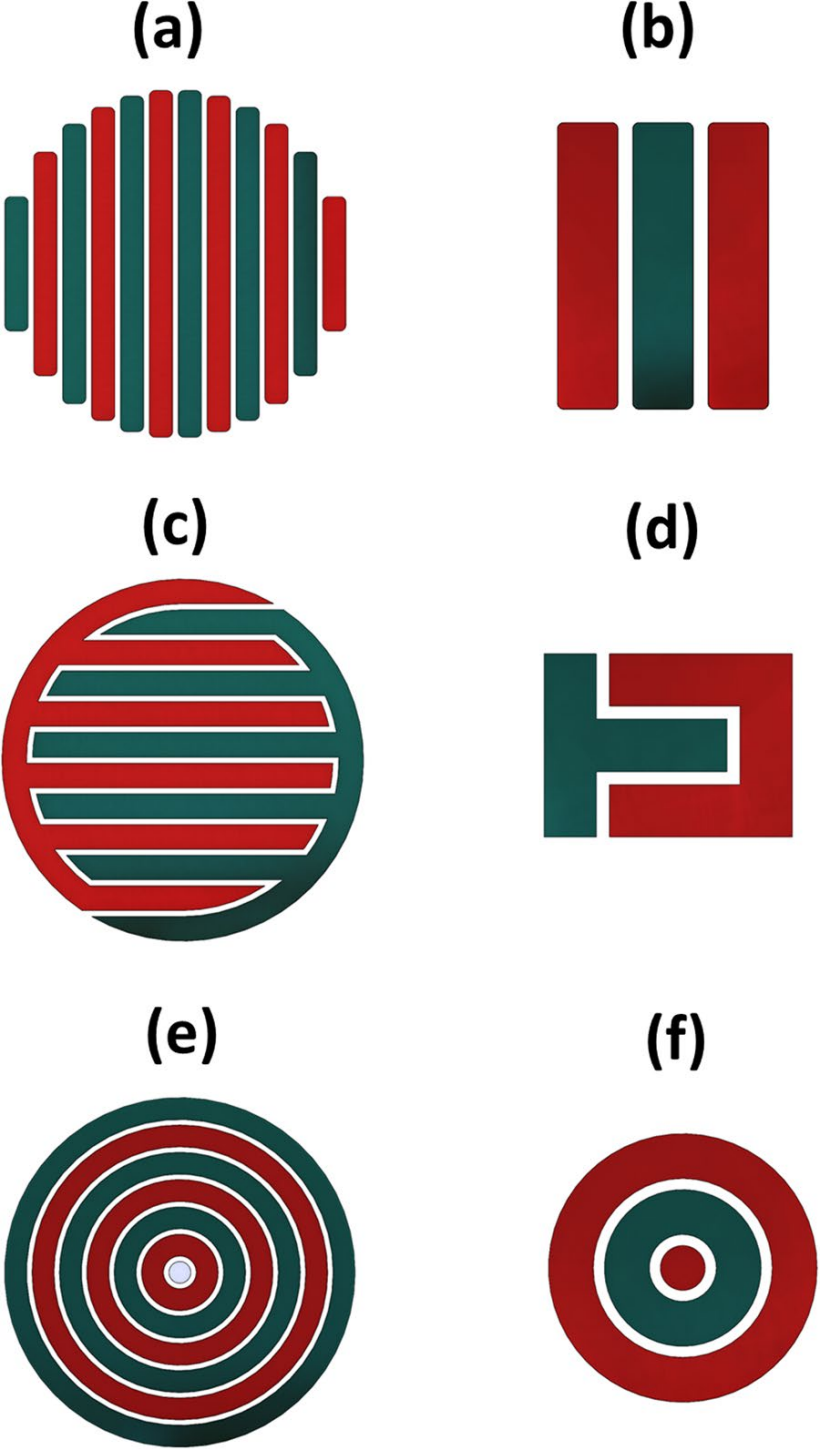
Configurations of electric powering of stimulators: (**a**) M-model of the striped pattern; (**b**) S-model of the striped pattern; (**c**) M-model of the interdigitated pattern; (**d**) S-model of the interdigitated pattern; (**e**) M-model of the circular pattern; (**f**) S-model of the circular pattern. Electrodes in red (anodes) were excited with *K*(1 − *cos*(*ωt*)); the remaining electrodes in green (cathodes) were grounded (0 V).

The electric excitations applied to drive the electrodes were defined to simulate an external power supply. Table 3 describes the chosen excitations parameters and the implemented configurations. Both low and high frequency excitations were analyzed. Frequencies of 14 Hz and 60 kHz were selected as positive osteogenic effects have already been demonstrated to be effective for these frequencies both *in vitro* (for MC3T3-E1 and other bone cell lines)^12,27–29^ and *in vivo*^30–35^ using capacitive stimulation (cosurface and parallel architectures). Sinusoidal waveforms are commonly used in electrical stimulation, both *in vitro* and *in vivo*^12,14,28–35^. Furthermore, recent studies emphasize that electrode excitations up to 10 V are able to up-regulate osteogenic stages (delivering stimuli throughout different daily exposure time and days of exposure) and are suitable for self-powered medical devices^12,14^. Finally, Fig. 4 shows the configurations defined to control the stimuli delivered to each region of the cell culture. All patterns and architectures were simulated considering anodes always surrounded by cathodes, so as to achieve a heterogeneous distribution of the electric field (section ‘Results’).

## Results

S-patterns were used to analyze *in silico* the impacts of cell confluence, cosurface architecture, electrodes geometry (thickness and width), gap size between electrodes and power excitation on the electric stimuli delivered to the cellular layer. A model validation was also performed to evaluate the ability to extrapolate stimulation results from S-models to M-models. Finally, the effects of low-frequency stimuli (14 Hz) delivered by stripped and interdigitated M-patterned stimulation apparatuses on MC3T3 pre-osteoblastic cells proliferation and differentiation were comparatively analyzed *in vitro*. The geometries of the M-patterned stimulation apparatuses used are as described in Table 1, although different electrode widths were used to explore their influence: 2 mm wide for the stripped pattern and 1 mm wide for the interdigitated pattern. Power excitation was applied as described in Table 3.

**Table 3:** Electric excitation parameters defined to power electrodes and related schematics.

Pattern	Waveform	Amplitude [V]	Frequency [Hz]	Schematic	Reference
Stripped M- and S-pattern	Sinusoidal *K*(1 − *cos*(*ωt*))	10 (*K* = 5)	14	Fig. 4a,b	^12, 27, 29, 52, 56, 57^
			60 k		^12, 28, 30– 35^
Interdigitated M- and S-pattern	Sinusoidal *K*(1 − *cos*(*ωt*))	10 (*K* = 5)	14	Fig. 4c,d	^12, 27, 29, 52, 56, 57^
			60 k		^12, 28, 30– 35^
Circular M- and S-pattern	Sinusoidal *K*(1 − *cos*(*ωt*))	10 (*K* = 5)	14	Fig. 4e,f	^12, 27, 29, 52, 56, 57^
			60 k		^12, 28, 30– 35^
Parallel M- and S-models	Sinusoidal *K*(1 − *cos*(*ωt*))	10 (*K* = 5)	14	Fig. 4g,h	^29, 43^
			60 k		^28, 44^

### Cell confluence influence on electric field stimuli

The stimuli distribution and dynamics along the cellular layer (low cell confluence condition; proliferation stage; *z* ∈ [0.5 0.51] [mm], where *z* is the vertical axis) or along the cellular tissue (full cell confluence condition; fully differentiated stage; *z* ∈ [0.5 0.52] [mm]) were found to be quite similar. Cross-correlations of nearly 100% and amplitude differences lower than 1% were perceived. Thereupon, only the stimuli delivered to bone cells throughout a fully differentiated culture will be analyzed in the following sections, i.e., along (*x*, *y*, 0.51) [mm] (in the *xy*-plan and a vertical coordinate *z* = 0.51 mm).

### Architecture influence on the electric field stimuli

**Figure 5:**
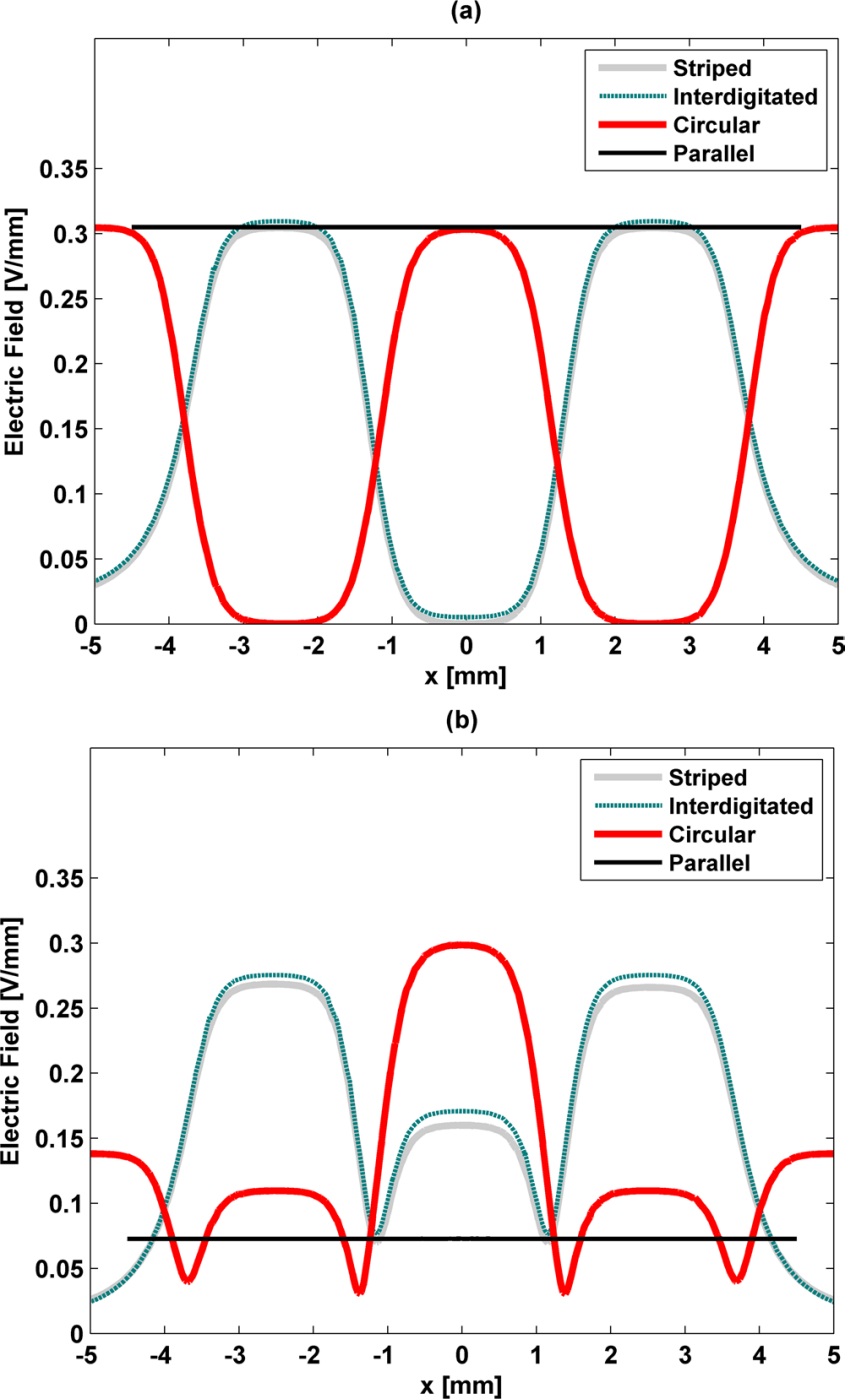
Electric field strength delivered by all patterns along (*x*, 0, 0.51) [mm], and dynamic behaviour in the point (0, 0, 0.51) using S-models for low frequency excitation (**a**) and high frequency (**b**).

**Figure 6:**
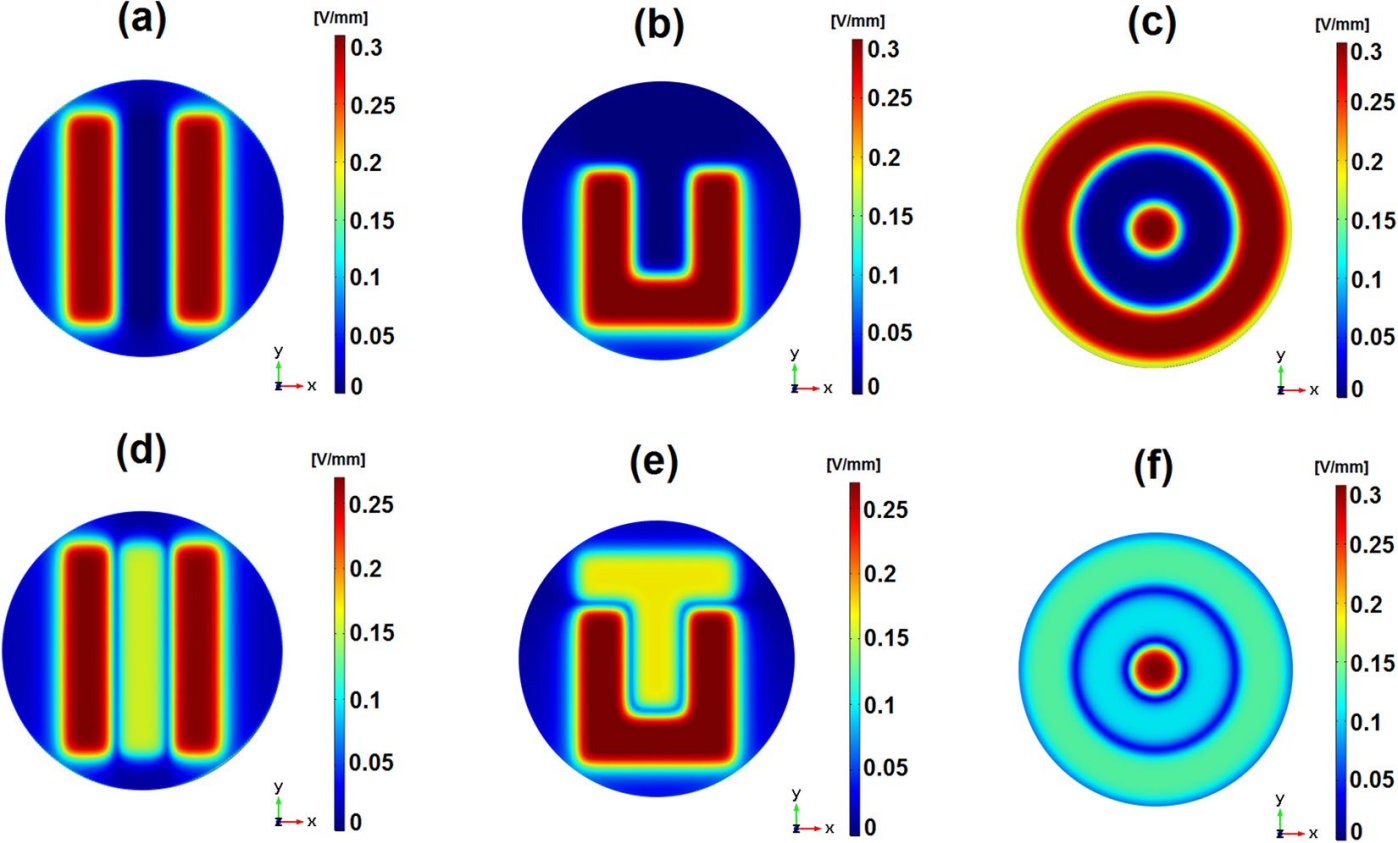
Electric field distributions using S-models for low frequency (**a**–**c**) and high frequency (**d**–**f**) excitations at *π* rad delivered by: (**a**,**d**) the stripped pattern along (*x*, *y*, 0.51) [mm]; (**b**,**e**) the interdigitated pattern along (*x*, *y*, 0.51) [mm]; (**c**,**f**) the circular pattern along (*x*, *y*, 0.51) [mm].

Frequency- and region-dependent electric field stimuli are delivered to osteoblastic cells, as shown by Figs 5 and 6. The following analysis firstly characterizes how cell cultures are stimulated by stripped and interdigitated patterns. Negligible differences are observed between the stimuli delivered by each pattern along the *x* axis (i.e., along (*x*, 0, 0.51) [mm]), either for low or high frequency excitations (Fig. 5). The heterogeneity of their electric field imposes maximum electric fields of 0.3 V/mm on the cellular layer above the positively charged electrodes (Figs 5 and 6a,b,d,e). In contrast, the lowest electric field is frequency-correlated: it occurs above the negatively charged electrodes for low frequency excitations (electric fields of approximately 0 V/mm), but above the gaps for high frequency excitations (electric fields of approximately 0.07 V/mm) (Figs 5 and 6a,b,d,e). Noticeably, the high-frequency electric fields stimulating cells are never reduced to zero (an electric field around 0.16 V/mm is observed above the negatively charged electrodes), as illustrated by Fig. 5b. The maximum stimuli magnitudes delivered by these stimulators are slightly higher (around 10%) for low frequency excitations as compared to high frequency excitations (Fig. 5). Differing stimuli distributions (along (*x*, *y*, 0.51) [mm]) are due to electrodes design (Fig. 6a,b,d,e). We can also infer from these results that the distributions of the stimuli generated by stripped and interdigitated architectures will increase in similarity with the increase of the length of the interdigitated stimulator electrodes.

Detailed analyses were also extended to the circular pattern. For low frequency excitations, the distribution and strength of the stimulus delivered by the circular pattern are similar to those delivered by both the stripped and interdigitated patterns along (*x*, 0, 0.51) [mm] (Fig. 5a). In this spectral region, similar heterogeneities are found, but shifted stimuli magnitudes are imposed (2.5 mm). Besides, the circular pattern exhibits a stimulus with differing distributions from the ones of the stripped and interdigitated patterns along (*x*, *y*, 0.51) [mm], as a result of its architecture (Fig. 6c,f). A pattern-dependent distribution is noticeable in high frequency stimulation provided by the circular pattern: maximum stimuli magnitudes around 0.3 V/mm are only predicted for the cellular layer above the innermost, positively charged, electrode. For the other electrodes besides the central one, the stimulus magnitude significantly decreases to 50–85% of the stripped and interdigitated patterns levels (Fig. 5b). Thus, this stimulator is able to provide geometrically nucleated stimuli. Similarly to the observed behavior of striped and interdigitated patterns for high frequency excitations, the circular pattern applies the lowest stimuli magnitude (of 0.03 V/mm) above the gaps.

Regarding the parallel architecture, homogeneous distributions are observed, as expected, and its stimulus magnitude at low frequency excitations is similar to the maximum stimuli magnitudes delivered by cosurface stimulators (Fig. 5a). Interestingly, its high-frequency stimuli only provide 45% and 53% of the average stimuli magnitudes delivered by the stripped/interdigitated patterns and the circular pattern, respectively (Fig. 5b).

### Influence of the electrode thickness on the electric field stimuli

*In silico* experiments were also conducted to understand how the stimuli delivery performance is altered as the electrode thickness is changed. M-models and S-models of all architectures were geometrically redesigned using electrodes with a much thinner thickness (0.1 mm). The remaining geometric variables were as Table 1 specifies. Results show that despite the fact that no influence is predicted for the electric field distribution when using low frequency stimuli (Fig. 5a vs 7a), remarkable differences are observed for high frequency excitations (Fig. 5b vs 7b). Stimuli distributions in high frequency are similar to the distributions in low frequency, but, surprisingly, the stimuli magnitudes are much higher than those delivered to cells by 1 mm thick electrodes (up to a 2.5-fold increase in the average and maximum magnitudes). The same occurs for the parallel architecture, with a 10-fold increase in stimuli magnitudes being observed.

**Figure 7:**
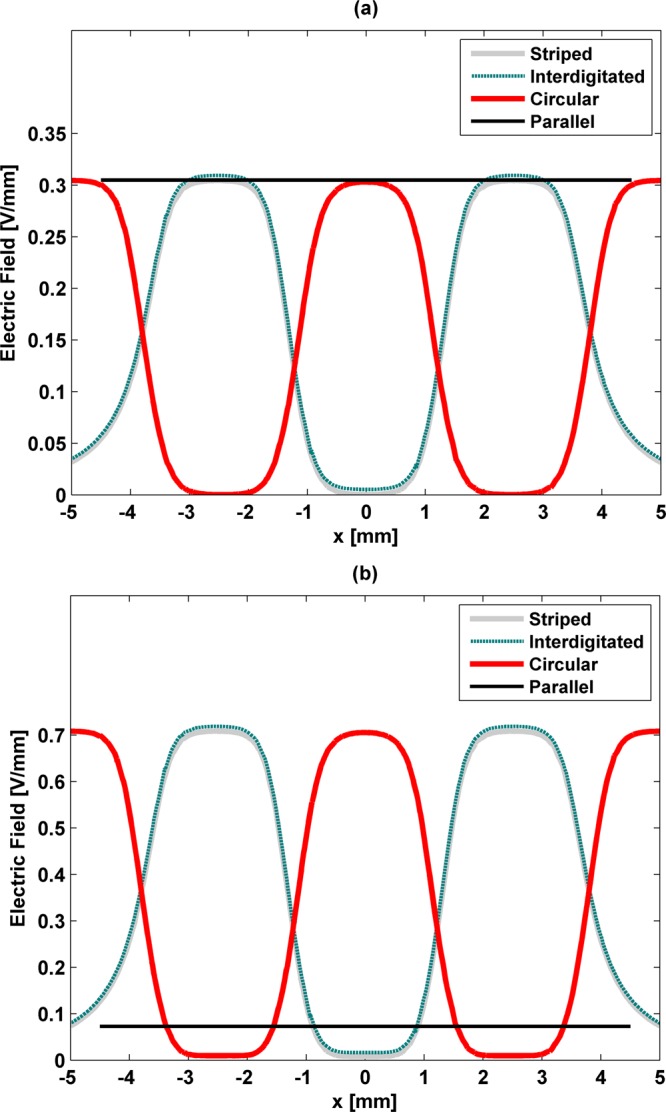
Stimuli delivered along (*x*, 0, 0.51) [mm] by all cosurface stimulators with 0.1 mm thick electrodes: using S-models for low frequency excitation (**a**) and high frequency (**b**).

### Influence of the electrode width on the electric field stimuli

**Figure 8:**
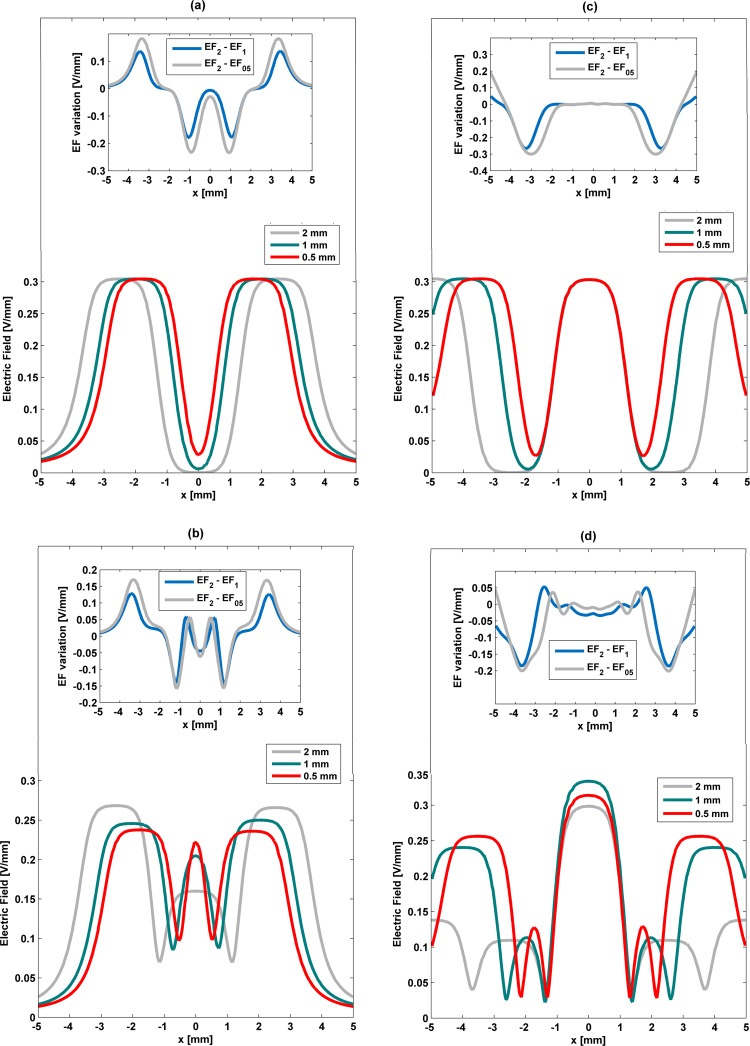
Influence of electrodes width (2 mm, 1 mm and 0.5 mm) on the stimuli delivered by striped and interdigitated patterns (**a**,**b**) and circular pattern (**c**,**d**) along (*x*, 0, 0.51) [mm] using S-models for low frequency excitation (**a**,**c**) and high frequency (**b**,**d**). EF_05_ - electric field for 0.5 mm electrodes width; EF_1_ - electric field for 1 mm electrodes width; EF_2_ - electric field for 2 mm electrodes width.

To highlight the influence of the electrodes width on cell stimulation, S-models of all architectures were redesigned using electrodes of different widths (2 mm, 1 mm and 0.5 mm). The remaining geometric variables were maintained constant and parameterized according to Table 1. Relevant phenomena occur on stimuli distribution with width variations (Fig. 8). Similar alterations are observed on the stimuli delivered by striped and interdigitated patterns for decreasing widths (Fig. 8a,c): (i) the changing effects mainly emerge in the same regions (|*x*| ≈ 1 mm and |*x*| ≈ 3.5 mm), regardless of the width decrease; (ii) similar stimuli magnitude differences occur for both low and high frequency stimuli; (iii) only increases on the stimuli magnitude difference occur as the width decreases.

These are the main results when the impact of changing the electrode width is predicted. However, a closer analysis also reveals a shortened region where the decrease in the electric field magnitudes is observed above the negatively charged electrodes, and the minimum magnitude that is higher (0.05 V/mm for 0.5 mm width; approximately zero magnitude otherwise). Indeed, null stimuli in this region do not occur when the electrodes’ width and gap between electrodes have similar dimensions (of 0.5 mm). Moreover, the width change only causes a horizontal shift in the stimuli distribution in cells placed above positively charged electrodes, while similar magnitudes are predicted. An increasing heterogeneity is observed when the high frequency stimulation is applied: (i) increased stimuli magnitudes on the cellular layer above negatively charged electrodes; (ii) increased stimuli magnitudes above the gaps; (iii) both decreased magnitudes and horizontal shifting of stimuli distributions on the cellular layer above positively charged electrodes (Fig. 8b). It is noteworthy that magnitudes around 0.23 V/mm above negatively and positively charged electrodes are ensured if both the electrodes width and gap between electrodes are defined as 0.5 mm.

Cell stimulation by the cosurface circular pattern also exhibits noteworthy phenomena for decreasing widths (Fig. 8c,d): (i) a frequency-dependent changing pattern is observed; (ii) the changing effects mainly emerge around the same region (|*x*| ≈ 3 mm for low frequency stimuli; |*x*| ≈ 2.5 mm and |*x*| ≈ 4 mm for high frequency stimuli); (iii) no significant magnitude differences are predicted.

In addition to these general results, one must also remark that the low frequency stimuli are not influenced by changes in the electrode width above the innermost electrode (Fig. 8c). Magnitude and waveform similarity is also observed above the outermost electrodes (0.3 V/mm). Conversely, differing behaviors occur above intermediate electrodes: the smaller the width, the shorter the decrease in the electric field magnitudes. Patterns with 0.5 mm wide and 0.5 mm gap are also able to deliver non-zero stimulation along the overall cell culture (c.a 0.05 V/mm minimum; Fig. 8a,c). A detailed investigation reveals that minimum electric fields occur: in the cell layer placed above a gap-electrode (negatively charged) interface for electrodes that are 1 mm wide, as well as in the cell layer placed above a gap, for 0.5 mm wide patterns. More complex distributions emerge from high frequency stimulation (Fig. 8d). Higher heterogeneities are expected as the electrode width decreases. Above the innermost electrodes, unexpected similar waveforms and differing magnitudes occur. Notice that a larger magnitude is delivered by the 1 mm wide pattern (0.33 V/mm). A significant increase on the magnitude stimuli (rates up to 85% of increase) occur for decreasing widths above the outermost electrodes, which come also along a shifting behavior (as observed for low frequency stimuli). Nevertheless, intermediate regions of the cellular layer become narrowed as width decreases. Although the analyses for this region confirm that minimum stimuli magnitudes (around 0.03 V/mm) are usually observed above gap-electrode interfaces, a minimum electric field is also surprisingly found in the cellular layer above a positively charged electrode for the 1 mm wide pattern.

### Influence of gap size between electrodes on the electric field stimuli

**Figure 9:**
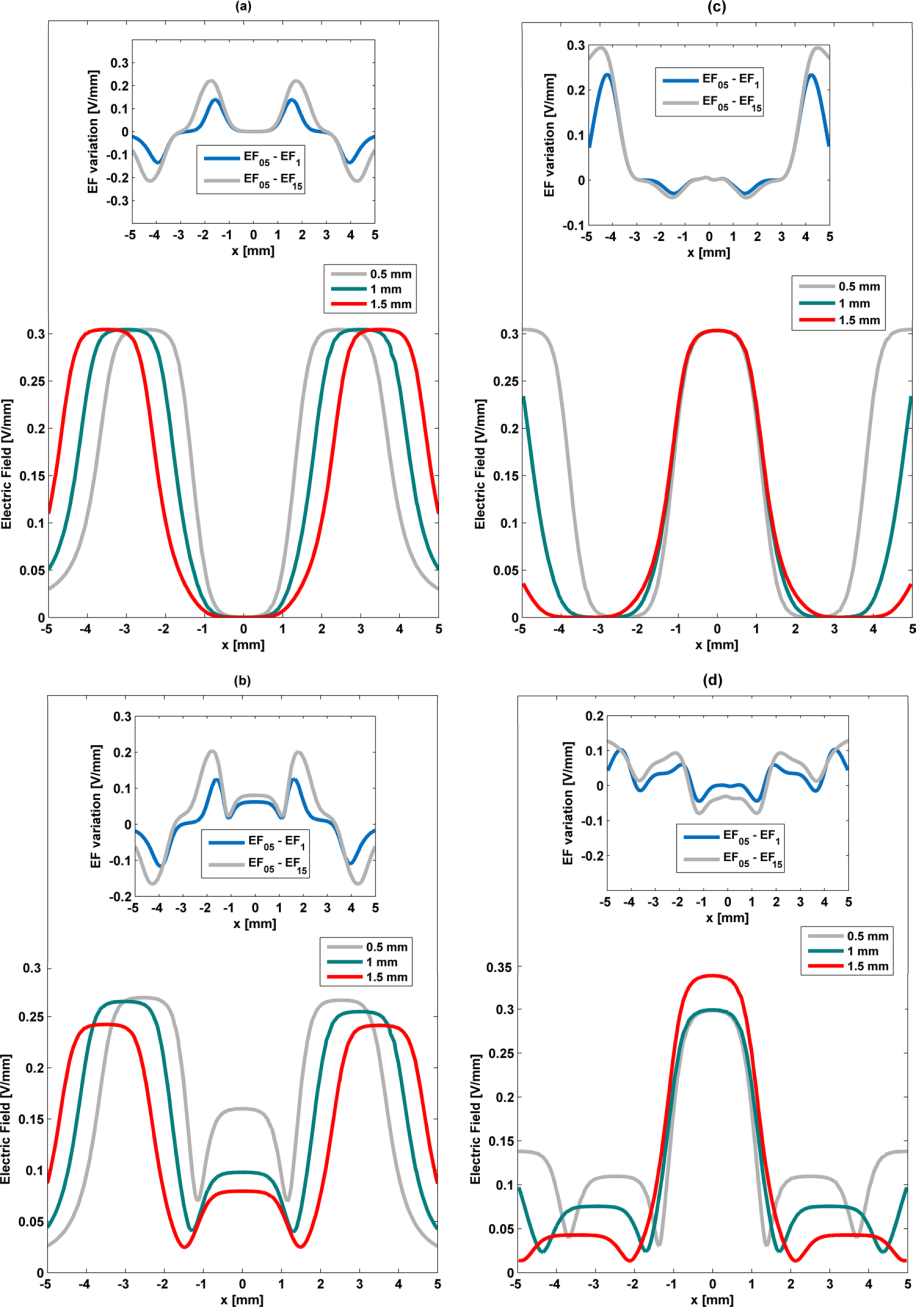
Influence of gap between electrodes (1.5 mm, 1 mm and 0.5 mm) on the stimuli delivered by striped and interdigitated patterns (**a**,**b**) and circular pattern (**c**,**d**) along (*x*, 0, 0.51) [mm] using S-models for low frequency excitation (**a**,**c**) and high frequency (**b**,**d**). EF_05_ - electric field for 0.5 mm gap size; EF_1_ - electric field for 1 mm gap size; EF_15_ - electric field for 1.5 mm gap size.

The influence of gap size between electrodes on cell stimulation was also examined by resizing gaps of the S-models to 1.5 mm, 1 mm and 0.5 mm. No change was defined to the remaining geometric variables (parametrization according to Table 1. Gap size variation conserves the stimuli distribution profiles but imposes shifts and alterations to their magnitude (Fig. 9). Nevertheless, similar changing patterns are also observed on the stimuli delivered by striped and interdigitated patterns for increasing gap sizes (Fig. 9a,c): (i) the changing effects mainly emerge in the same regions (|*x*| ≈ 2 mm and |*x*| ≈ 4 mm), regardless the gap size increase; (ii) similar stimuli magnitude differences are observed for low and high frequency stimulation; (iii) only increases on the stimuli magnitude difference occur as the gap size increases.

These results emphasize that the gap size influence is similar to the electrode width influence (only differing by their signs) for both striped and interdigitated patterns Figs 8a,c and 9a,c). Detailed analyses reveal that low frequency stimuli distributions are horizontally shifted in the cellular layer above positively charged electrodes (Fig. 9a). It is true that similar stimuli magnitudes (0.3 V/mm) are found in this same region, but one must also emphasize the extended region where the electric field magnitudes decrease (meaning, larger distribution areas stimulated below 0.3 V/mm) as the gap size increases. The gap size does not influence the distribution pattern along the cellular layer for high frequency stimulations, instead it alters the stimuli magnitude and slightly shifts the distribution (Fig. 9b). Increasing the gap size results in the following effects: (i) decreasing magnitudes and horizontally shifted waveforms above positively charged electrodes; (ii) decreasing magnitudes above negatively charged electrodes, as well as above gaps; (iii) lower and slightly shifted minimum stimuli magnitudes; (iv) slightly larger distribution of constant stimuli in the cellular layer above negatively charged electrodes.

The gap size also influences the stimuli delivered by the circular pattern. An overall analysis highlights that, as the gap size increases, cell stimulation is mainly focused above the innermost electrodes (Fig. 9c,d). Other general patterns are also perceived (Fig. 9c,d): (i) a frequency-dependent changing pattern is observed; (ii) the changing effects mainly emerge around the same region for low frequency stimulation (|*x*| ≈ 4 mm); (iii) for high frequency stimulation, increases on the stimuli magnitude difference are established as the gap size increases. Under low frequency stimulation, negligible influence is exerted above the outermost electrodes and subsequent gap (only slightly different distributions bellow 0.05 V/mm are distinguishable; Fig. 9c). In addition, for increasing gap sizes there is an increase in non-stimulated areas above the outermost electrodes. The delivery of high frequency stimulation implies negligible influence of gap sizes up to 1 mm on both the stimuli magnitude and waveform above the innermost electrodes. However, larger gaps will provide magnitude amplification (rates up to 13% increase are expected for 1.5 mm gap), as evidenced by Fig. 9d. The same cannot be stated about the stimulation delivered to cells above the outermost electrodes: magnitudes decrease in the same level as the gap size increases. Notice that for 1.5 mm gap the cellular layer above the outermost electrodes is sparsely stimulated (with electric fields lower than 0.05 V/mm).

### Influence of power excitation on the electric field stimuli

**Figure 10:**
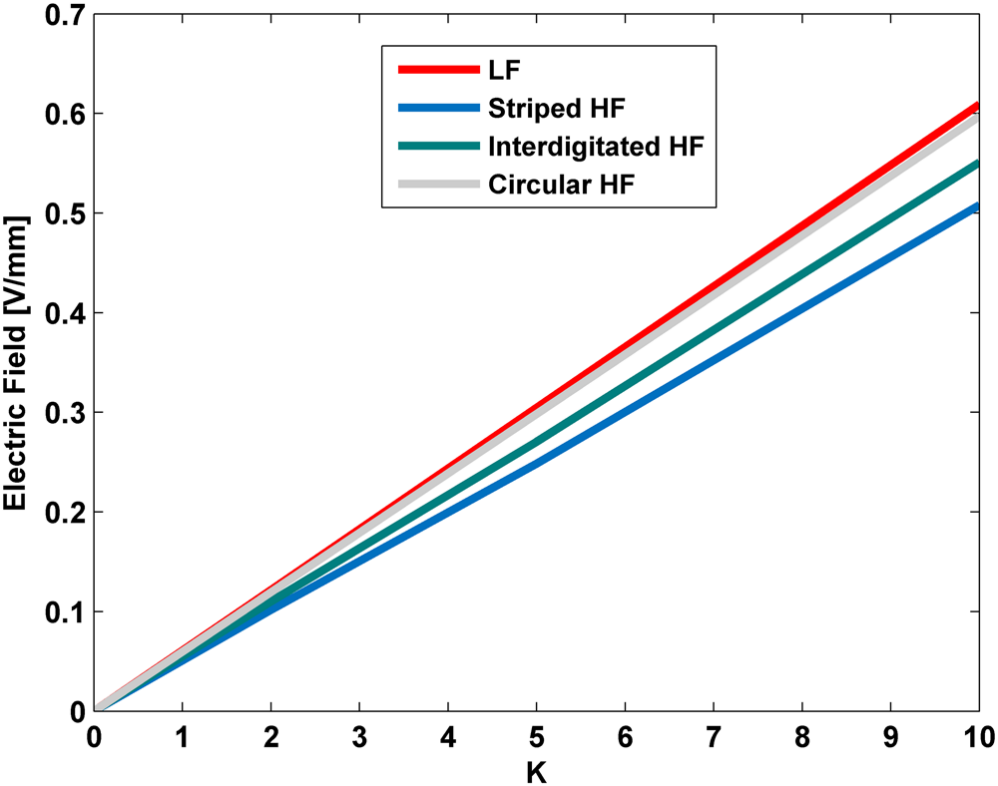
Influence of the power excitation (K = 10 for 20 V) on the electric stimulation along (*x*, 0, 0.51) [mm] using S-models. LF - similar stimuli delivered by all patterns.

Linear interrelationships occur between power excitations supplying electrodes and the stimuli magnitudes delivered to cells along the overall cell layer, as Fig. 10 illustrates. This proportionality was observed for power excitations up to 20 V of amplitude (*K* = 10), regardless of the cosurface pattern and frequency. Similar slopes *E* [V/mm]/*V* [V] (≈0.057) are observed.

### Model validation

**Figure 11:**
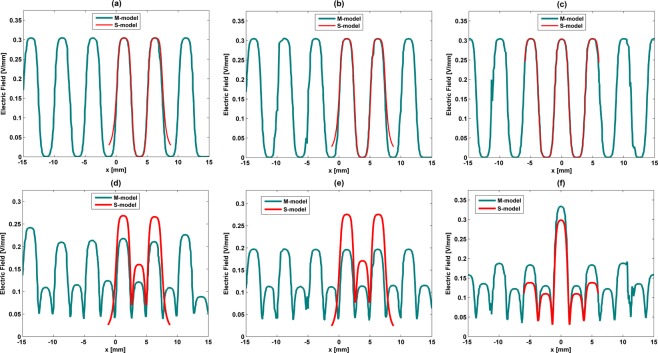
Comparison between electric field distributions using both S-models and M-models for low frequency (**a**–**c**) and high frequency (**d**–**f**) excitations using the striped pattern (**a**,**d**), interdigitated pattern (**b**,**e**) and circular pattern (**c**,**f**): stimulators comprising electrodes with 1 mm thick.

**Figure 12:**
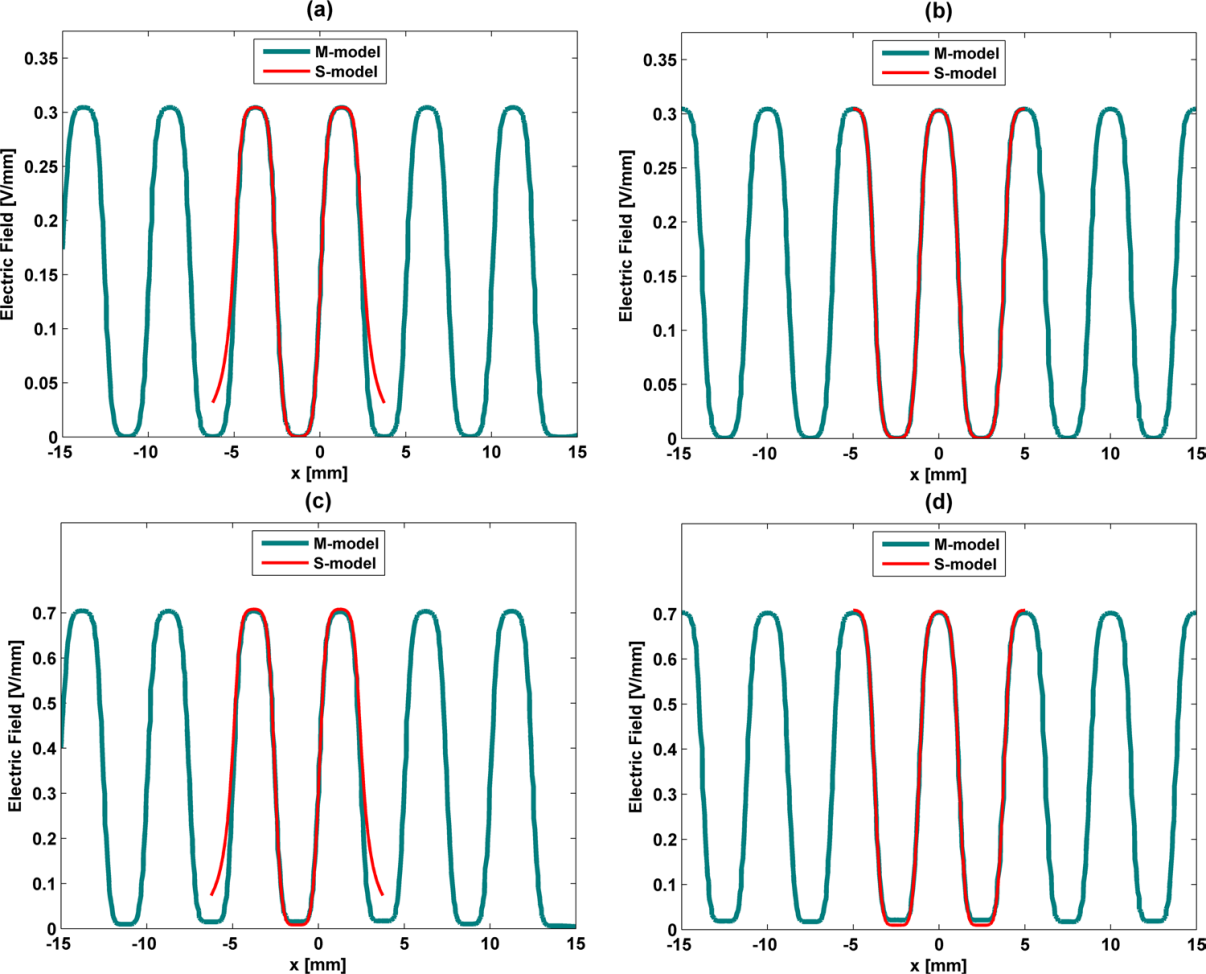
Comparison between electric field distributions using both S-models and M-models for low frequency (**a**,**b**) and high frequency (**c**,**d**) excitations using the striped and interdigitated patterns (**a**,**c**) and circular pattern (**b**,**d**): stimulators comprising electrodes with 0.1 mm thick.

Electric stimuli provided by S-models and M-models were compared for validation purposes. Validation results highlight that low frequency stimulation delivered by M-patterns, parametrized as stated in Table 1 (1 mm thick electrodes), can be accurately predicted by the S-models (Fig. 11a–c). Waveform cross-correlations of approximately 99% were observed when M-models and S-models of stripped, interdigitated and circular patterns were compared. Although very high waveform cross-correlations were also obtained for high frequency stimulation (approximately 96.5%), lower magnitudes are expected when M-patterns of striped and interdigitated stimulators are used to perform cell stimulation (overall magnitude offsets of approximately 0.05 V/mm and 0.075 V/mm, respectively) and higher magnitudes for M-patterns of circular stimulators (overall magnitude offset of approximately 0.03 V/mm), as illustrated by Fig. 11d–f. Much better validation outcomes were computed when the electrodes thickness is thinned (Fig. 12). Waveform cross-correlations around 99% are found for both low (Fig. 12a,b) and high frequency stimulation (Fig. 12c,d), as well as similar stimuli magnitudes, if cosurface stimulators comprise 0.1 mm thick electrodes.

### Influence of the stimulators architecture on *in vitro* osteoconductive effects

**Figure 13:**
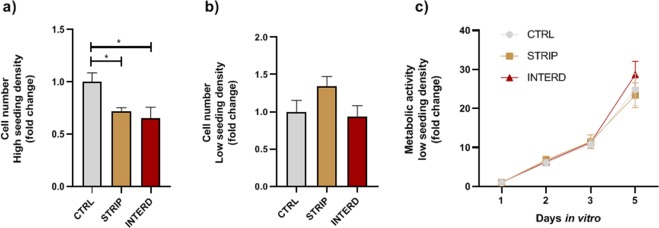
Proliferation and viability of MC3T3 cells cultured in the absence of stimuli (CTRL), or daily exposed to low-frequency electric stimulation with two electrode patterns (stripped [STRIP] and interdigitated [INTERD]). Initial cell proliferation was assessed 24 h after seeding in (**a**) high density (1 × 10^4^ cells/cm^2^) and (**b**) low density (1 × 10^3^ cells/cm^2^). (**c**) Metabolic activity was measured over 5 days *in vitro* using the resazurin colorimetric assay. *p < 0.05.

*In vitro* biological assays were conducted using stripped and interdigitated M-models with different electrode widths and MC3T3 pre-osteoblastic cells. First, cellular proliferation was measured 24 h after cells exposure to electrical stimulation of low frequency (Fig. 13). Under these conditions, the effects of the delivered electric fields on cell proliferation seem to be influenced both by cell seeding density and electrode configuration. In a high confluent density, a statistically significant decrease in proliferation is observed for both electrode configurations (Fig. 13a). On the other hand, in a pre-confluent low density, the stripped-based stimulator induces a slight increase in cell proliferation over control, although with no statistical significance (Fig. 13b). These early (24 h) alterations in cell proliferation are not accompanied by a decrease in cell metabolic activity (Fig. 13c). Indeed, the resazurin assay of a low-density starting culture revealed no differences in the metabolic activity of cells between stimulated and non-stimulated cells, regardless of electrode pattern (Fig. 13c). Nevertheless, a slight enhance in cell metabolism is observed for the interdigitated pattern following 5 days *in vitro* (DIV) stimulation.

**Figure 14:**
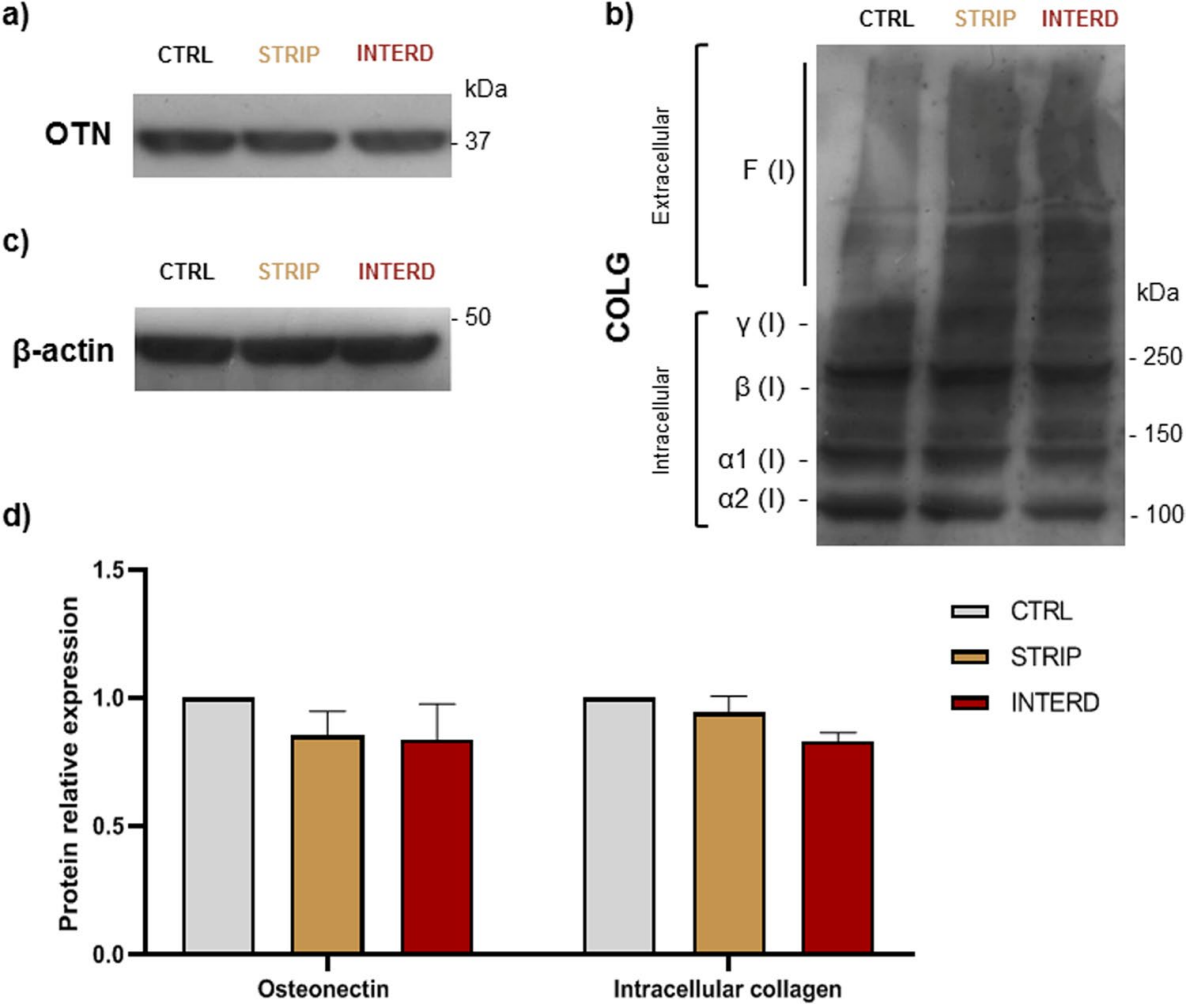
Intracellular matrix maturation markers in MC3T3 cells cultured in the absence of stimuli (CTRL), or daily exposed to low-frequency electric stimulation with two electrode patterns (stripped [STRIP] and interdigitated [INTERD]) at 7 DIV. Immunoblot analysis of (**a**) osteonectin (OTN); (**b**) intracellular and extracellular collagen-I forms: unprocessed and processed *α* 1(I) and *α* 2(I) procollagen monomeric chains (130–160 kDa); *β*(I), procollagen dimeric forms (≈270 kDa); *γ*(I), procollagen trimeric forms (≈400 kDa); F(I), collagen fibrils of high molecular weight that have difficulty entering the SDS-PAGE gel; (**c**) *β*-actin, used as loading control. Migration of molecular weight markers is indicated to the right. Of note, bands are presented as they are in the gel/blot/film, with no regrouping of cropped parts. (**d**) Relative quantification of osteonectin and intracellular collagen-I levels, presented as fold increase over CTRL values.

**Figure 15:**
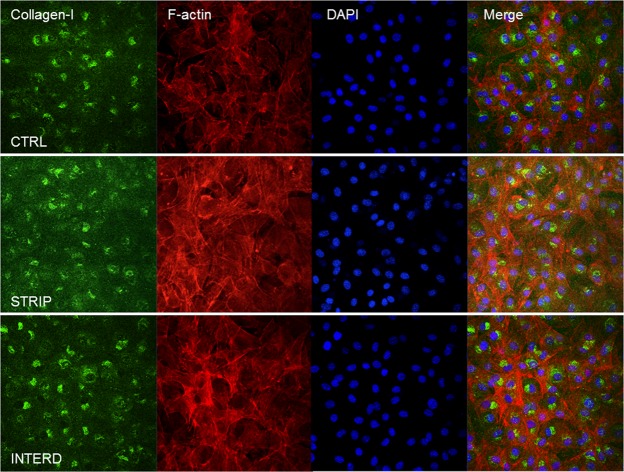
Confocal microscopy analysis of MC3T3 osteoblasts cultured for 28 DIV in the absence of stimulus (CTRL) or upon daily stimulation with two electrode patterns (stripped [STRIP] and interdigitated [INTERD]). Cells were probed for type-I collagen (green fluorescence), DAPI (blue nuclear staining), and filamentous actin (F-actin, probed with red-labeled phalloidin). Scale bar = 50 *μ*m.

To assess matrix maturation upon electrical stimulation with stripped and interdigitated M-patterns, the levels of osteonectin and type-I collagen were analyzed by immunoblot following 7 days of stimulation (Fig. 14). Intracellular levels of both osteonectin and collagen-I proteins do not alter, and may even slightly decrease under stimulation (Fig. 14). This is particularly true for collagen-I monomers and dimers, and seems to be due to extracellular deposition of higher molecular weight forms of collagen-I. Indeed, intracellular collagen trimers [*γ*(I)] and extracellular fibrils [F(I)] that do not enter the resolving gel^36^, appear increased upon stimulation (Fig. 14). This is similar to what we have reported before for the stripped pattern^12^. Further, when the collagen staining is assessed in cells stimulated for 28 days, higher collagen staining can be observed in the extracellular matrix of electrically-stimulated cultures (Fig. 15, green diuse staining). This higher matrix deposition seems not to come from higher collagen secretion, as the profile of secreted collagen-I remains unchanged (Supplementary Fig. S1).

**Figure 16:**
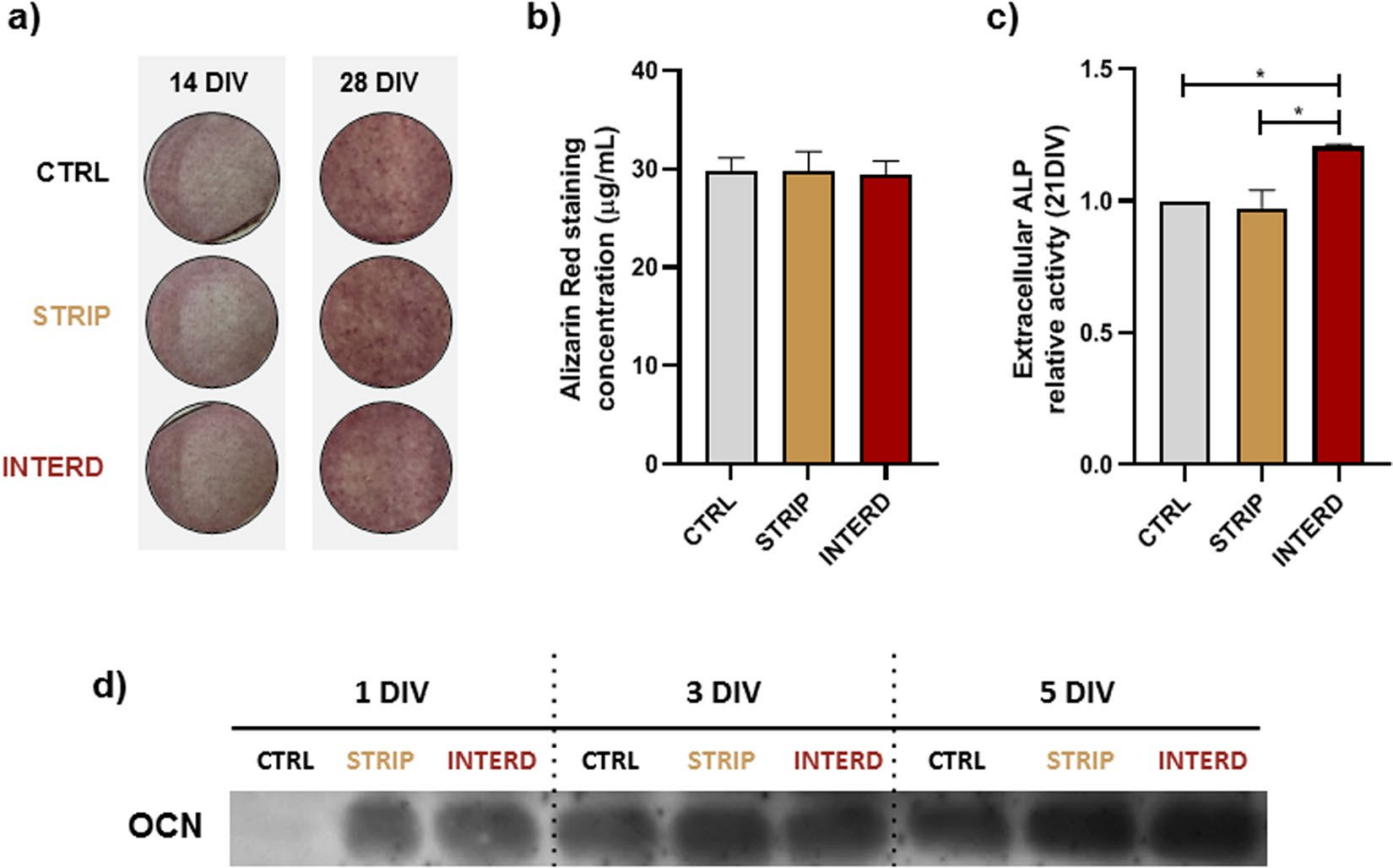
Analyses of the matrix mineralization markers in MC3T3 cells cultured in the absence of stimulus (CTRL) or upon daily stimulation with two electrode configurations (stripped [STRIP] and interdigitated [INTERD]). (**a**) Photographs of calcium deposits in cultured cells at 14 and 21 DIV, red stained by the Alizarin Red S (ARS) dye. (**b**) Quantification of the ARS staining at 21 DIV. (**c**) Relative secretion of ALP into the cells conditioned medium (expressed as fold increases over CTRL levels) at 21 DIV. (**d**) Immunoblot analysis of secreted osteocalcin (OCN) into the cells’ conditioned medium, from 1 to 7 DIV; loading control is presented in Supplementary Fig. S1. DIV - days *in vitro*. Of note, bands are presented as they are in the gel/blot/film, with no regrouping of cropped parts.

The levels of matrix mineralization were assessed with the calcium-staining dye Alizarin Red (ARS). As expected, ARS staining increased from 14 to 28 DIV for all conditions (Fig. 16a), but no differences were observed for the different conditions at 28 DIV (Fig. 16b). Nevertheless, the levels of active alkaline phosphatase (ALP), a glycoprotein that increases medium phosphate levels by hydrolysing organic phosphates and a marker for osteoblast activity and differentiation^37,38^, were influenced by the electrode architecture (Fig. 16c). While stimulation for 21 DIV with the stripped pattern did not influence the ALP levels, as previously reported by us^12^, stimulation using the interdigitated pattern increased the levels of secreted ALP to 1.21 ± 0.01 (Fig. 16c). Furthermore, electric stimulation with both architectures, but particularly with the interdigitated pattern, increased the profile of osteocalcin secretion over time (Fig. 16d).

## Discussion

The therapeutic potential of non-drug strategies, mainly those performed by biophysical signals (electric, acoustic, thermal, mechanical, etc.), has been deeply explored for the treatment and prevention of multiple conditions, such as musculoskeletal and neurological, disorders among many others. The widespread clinical and research applications based on electromagnetic stimulation have emphasized its ability to provide personalized medical care. Recent research findings have revealed unique insights into novel architectures to deliver customized stimuli to target tissues. This is of upmost importance when considering, for example, that the regulation of neural activity by electric stimulation of specific brain regions requires the personalization of the stimulators’ electrodes^3,18,39^. The proposal for a new era of intracorporeal bone implants also envisages personalized electromagnetic stimuli delivery to bone tissues and related electrodes customization^12^. These are instrumented active implants comprising electromagnetic stimulation systems and osseointegration monitoring systems that will ultimately be controlled by clinicians. Such active implants hold potential to induce positive osteogenic responses at different stages of bone remodeling, such that the peri-prosthetic bone can be controlled to minimize surgical revisions caused by adverse bone remodeling and loss^12,23–25,40–42^. The superior performance of novel and innovative medical devices requires personalized stimuli waveform, magnitude and frequency, among other stimulation parameters, which in turn demand personalized excitations of electrodes (waveform, magnitude, frequency, etc.) and personalized stimulators architectures and electrodes configurations. Cosurface electrode architectures are suitable both for extra- and intracorporeal therapies and, according to recent advances, they may achieve superior performances as, in a near future, they may impose optimized spatio-temporal tissue dynamics^12^. Nevertheless, there is a lack of studies related to the impact of the electrodes configurations on the delivery of controlled electric stimuli to target tissues.

The influence of the stimulator architecture and geometry on stimuli distribution is provided, for the first time, in this study. Included are analyses with a cosurface circular pattern that, to the knowledge of the authors, has never been studied for biomedical purposes. These analyses were firstly considered for cell culture *in vitro* experiments, even though further investigations must be conducted for much more complex bone structures, such as trabecular and cortical structures comprising liquid, organic and mineral phases. The interplay between stimuli parameters and their osteoconductive responses must be primarily addressed as preliminary performance analyses of these new capacitive technologies for sophisticated therapies. Computational models were developed according to approximate apparatuses, namely using approximately homogeneous cellular phases during the proliferation stage (only composed by non-confluent MC3T3 cells), and approximately homogeneous cellular tissues during the differentiation stage (composed by confluent MC3T3 cells and a type-I collagen matrix). These assumptions were undertook because of the low concentration of the physiological culture medium around cells, as well as due to the non-significant dielectric change of the cellular medium throughout proliferation and differentiation stages^12^. Further studies must also be carried out to include stimuli analyses throughout the mineralization stage, which will require more complex dielectric structures, as an inhomogeneous cellular tissue composed of both organic and extracellularly deposited inorganic components must be considered. Regarding its research potential, notice that the method here proposed can be applied to achieve major findings concerning stimuli distribution throughout many other biological tissues.

The stimuli delivered by these cosurface architectures are the same order-of-magnitude as stimuli with ability to induce positive osteoconductive responses at different stages of bone remodeling^12,28,43,44^. As differing stimuli distributions are delivered to the cellular layers for different excitations imposed to the electrodes and for different stimulator designs, stimuli personalization requires voltage- and frequency-dependent optimization (external customization), and architecture- and geometry-dependent optimization (internal customization). According to results highlighted in section ‘Results’, all these parameters must be carefully addressed for effective biomedical therapies. Significant changes on electric stimuli distributions are obtained as the stimulation frequency is significantly changed, unless very thin electrode thicknesses are designed. In this case, the thickness influence is well perceived on the stimuli magnitudes. The thickness of the electrodes is of utmost importance for the biodevices as the sophistication trend imposes volume minimization of devices and optimization of their functions. Although all stimulation patterns here analyzed allow for the application of equal power excitations (as they use similar number of electrodes), stimuli distributions exhibited by the cosurface circular pattern are clearly distinct from those provided by stripped and interdigitated patterns. The analysis of the influence of the electrode architecture emphasizes the importance of using computational models to predict the stimuli distributions. Although alterations on the width of the electrodes and gaps between them do not impose very differing stimuli distribution profiles, results are not reduced to shifts in their magnitudes: other non-negligible effects must be considered. On the other hand, the impact of additional electrodes can be easily predicted using thin electrode thicknesses.

Biological experiments reported in this study promisingly demonstrate that cosurface stimulation can enhance osteoconductive processes, as recent and preliminary research findings already highlighted^12,14^. The architectures here tested were able to induce positive responses, mainly in the osteoblasts differentiation phase. Further, the effects were similar for both stripped and interdigitated architectures, as expected since the electric stimuli delivered by those architectures are generally similar (Fig. 6). Nevertheless, slightly better osteodifferentiation results were observed for the interdigitated stimulator, potentially due to differences in the electric field stimuli distributions delivered by stripped and interdigitated patterns resulting from the different electrode widths (Fig. 8). Future research will be extended to the influence of high frequency stimulation in *in vitro* assays, and will include the circular pattern. The observed success in terms of osteoconductive responses provides strong motivation and justification for studying the ability of these architectures to stimulate more complex structures, such as trabecular bone structures, and other type of tissues, as neural ones.

## Conclusion

This research work is focused on the ability of three cosurface capacitive stimulation systems (stripped, interdigitated and circular architectures) to provide effective osteoconductive stimuli to target tissues, such that they can be embedded in the future into multifunctional bioelectric implantable medical devices. The following core findings can be highlighted:

1. All capacitive cosurface stimulators analyzed are able to deliver osteogenic stimuli;
2. The influence of cell culture confluence is negligible;
3. Electric field stimuli is architecture-, frequency- and region-dependent;
4. Electric stimuli delivered by stripped architectures are quite similar to the one delivered by interdigitated architectures;
5. Low frequency stimuli distributions are similar to high frequency stimuli distributions for very thin electrodes (0.1 mm);
6. High frequency stimuli magnitudes are 2-fold higher than low frequency stimuli magnitudes for very thin electrodes (0.1 mm);
7. The influences of electrodes’ width and gap size are similar for stripped and interdigitated architectures: differences in the stimuli emerge around the same regions; differences in the stimuli magnitude are similar for both low and high frequency excitations; differences in the stimuli magnitude are expected to increase as the width decreases/gap size increases.
8. Simplified models of stimulators can be used to easily predict the impact of additional electrodes on electric stimulation if thin electrode thicknesses are used.
9. Some electrode architecture-dependent influences, determined by modeling assays, can be validated in *in vitro* assays biomedical experiments.

## Methods

### Numerical simulation

All M- and S-models were developed using the AC/DC module of COMSOL Multiphysics (v. 4.4, COMSOL). This computer simulation tool has been successfully used to analyse electromagnetic stimulation of biological tissues^12,15,45,46^. All domains were defined as homogeneous and isotropic, and were tessellated by fine 3D meshes of tetrahedral linear elements of second order (Delaunay method). Mesh refinement and dimensioning of the ‘Air’ domain were conducted by convergence analysis (2% error as stop criterion). The homogeneous Neumann condition was imposed to interior boundaries. Electrodes were powered by using terminal (anodes) and ground (cathodes) nodes. External boundaries were electrically isolated. Electromagnetic fields were simulated by using the ‘Magnetic and Electric Fields’ as the physics interface since it allows to solve the Maxwell’s equations in the frequency-domain, as expressed by Eqs (1) to (8).

1 $$\nabla \cdot {\bf{J}}=0$$

2 $${\bf{E}}=-\,\nabla \cdot V-\frac{d{\bf{A}}}{dt}$$

3 $$\nabla \times {\bf{H}}={\bf{J}}$$

4 $$B=\nabla \times {\bf{A}}$$

5 $${\bf{J}}=\sigma {\bf{E}}+\frac{d{\bf{D}}}{dt}$$

6 $${\bf{D}}={\varepsilon }_{0}{\varepsilon }_{r}{\bf{E}}$$

7 $${\bf{B}}={\mu }_{0}{\mu }_{r}{\bf{H}}$$

8 $${{\bf{n}}}_{2}\cdot ({{\bf{J}}}_{1}-{{\bf{J}}}_{2})=0$$

where: **E** - electric field intensity [V/m]; **D** - electric displacement [C/m^2^]; **H** - magnetic field intensity [A/m]; **B** - magnetic flux density [T]; **J** - current density [A/m^2^]; **A** - magnetic vector potential [Vs/m]; *σ* - electrical conductivity [S/m]; *ε*_0_ - permittivity of vacuum (8.85 × 10^−12^ [F/m]); *ε*_*r*_ - relative permittivity; *μ*_0_ - permeability of vacuum (4 *π* × 10^−7^ [H/m]); *μ*_*r*_ - relative permeability; *V* - electric scalar potential [V]; **n**_2_ - outward normal from medium 2 at interfaces between two media **J**_1_ and **J**_2_; **J**_*n*_ - current densities of medium *n* [A]. The linear FGMRES solver was used to provide fast convergence and computing robustness. M- and S-models were computed in a workstation (Precision T5500, Dell) with 12 CPUs of 2.4 GHz and 24 GB RAM.

### Analysis of electric field data

**Figure 17:**
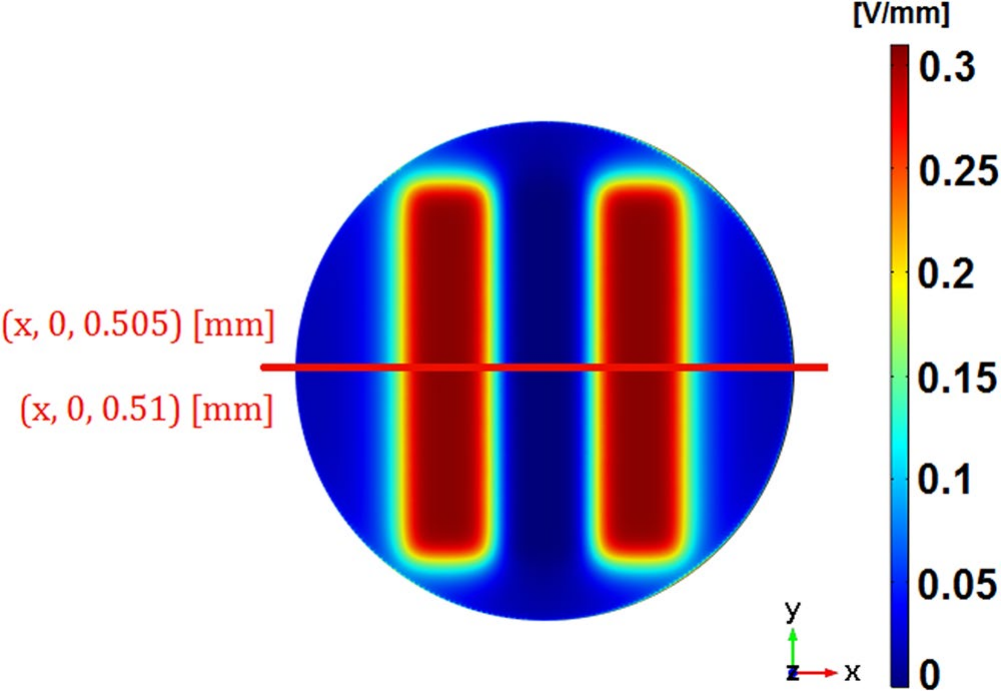
Electric field along (*x*, *y*, 0.505) [mm] and (*x*, 0, 0.505) [mm] for stimuli analysis throughout the proliferation stage, as well as along (*x*, *y*, 0.51) [mm] and (*x*, 0, 0.51) [mm] for the differentiation stage. Results illustrated in this figure were selected from section ‘Results’ just to highlight how data analysis was conducted.

The electric field was analysed along a *xy*-plan in a vertical z-coordinate corresponding to the cellular layer/tissue midpoint, i.e., along (*x*, *y*, 0.505) [mm] for the stimuli delivered throughout the proliferation stage, and (*x*, *y*, 0.51) [mm] for the differentiation stage (notice that the origin plan (*x*, *y*, 0) match the upper boundary of electrodes). Furthermore, detailed analysis was conducted by observing the stimuli along the line *y* = 0 [mm], specifically along (*x*, 0, 0.505) [mm] and (*x*, 0, 0.51) [mm], as clarify Fig. 17. Section ‘Results’ highlight the electric field observed at *π* rad, although its dynamic behaviour was also analysed.

### Stimulation apparatuses for *in vitro* experiments

Stripped and interdigitated stimulators were implemented according to M-models described in Table 1. Different thicknesses were used: 1 mm thick electrodes for the stripped pattern; 0.1 mm thick electrodes for the interdigitated pattern. Excitations to power stimulators were configured using a real-time application that was developed using Simulink (v. 7.3, Mathworks) and the Real Time Workshop (v. 7.3, Mathworks) and run using the Real Time Windows Target (v. 3.3, Mathworks) kernel. Excitations were generated by an IO card (MF 624, Humusoft). Stimulators were powered by excitations as described in Table 3. The overall schematics of the stimulation system is illustrated in Fig. 2.

### Cell culture and cell seeding

The osteoblastic MC3T3-E1 cells (CRL-2593, ATCC, Barcelona, Spain) were maintained at 37 °C in a 5% CO_2_ humidified atmosphere with 2 mM L-glutamine-containing Minimum Essential Medium alpha (MEM *α*) in Eagle’s solution, supplemented with 2.2 g.L^−1^ NaHCO_3_, 10% (v/v) foetal bovine serum and 1% (v/v) antibiotic-antimycotic solution (10,000 units.mL^−1^ penicillin; 10,000 *μ*g.mL^−1^ streptomycin; 25 *μ*g.mL^−1^ Amphotericin B; Gibco BRL, Invitrogen, USA). Cellular expansion was performed using 0.05% trypsin/EDTA (Gibco BRL). *In vitro* 14 Hz electric stimulation was carried out for 28 days (4 h/day) in a CO_2_ instrumented incubator (Galaxy 14S, New Brunswick Scientific)^12^.

### Proliferation and metabolism assays

The Trypan Blue (Sigma-Aldrich, UK) membrane exclusion assay was used to assess cell proliferation. Briefly, 1 × 10^4^ and 1 × 10^5^ cells were plated into 35 mm dishes with 1 mL fresh media. Upon 24 h in culture at 5% CO_2_/37 °C under electrical stimulation, the number of viable cells was directly scored as previously^12^ and presented as fold changes over control non-stimulated cells. The resazurin colorimetric assay was used to determine the effects of striped and interdigitated electrodes on cell metabolism/viability^47^. Briefly, at 1, 2, 3, and 5 DIV cells were incubated for 4 h with fresh medium containing 10% of a resazurin stock solution (0.1 mg.mL^−1^ resazurin salt, Sigma-Aldrich, UK) in phosphate-buffered saline (PBS) (Thermo Fisher Scientific, USA). Resazurin reduction was monitored at 570 and 600 nm (Infinite 200 PRO, Tecan). The OD 570/OD 600 nm ratio of the blank was subtracted to the OD 570/OD 600 nm ratio of each condition, and the result presented as fold change over control non-stimulated levels at 1 DIV.

### Alkaline phosphatase (ALP) activity

Secreted ALP activity was determined using *ρ*-nitrophenyl phosphate (Calbiochem, Merck, Germany) as the enzyme substrate. Conditioned media of 21 DIV stimulated cells were collected, and triplicate 20 *μ*L aliquots incubated for 1 h in the dark at 37 °C, with 200 *μ*L of the ALP alkaline substrate solution. The O.D. of enzymatic products was measured at 405 nm (Infinite 200 PRO) and presented as fold change over control non-stimulated levels.

### Immunoblot evaluation of osteoblast differentiation markers

Expression of secreted and synthesized protein markers was determined in conditioned medium and cell lysates, respectively. Conditioned media was collected to 10% SDS-containing microtubes at several DIV and cell lysates were directly harvested at 7 DIV with 1% SDS and sonicated for 30 sec. All collected samples were boiled and the protein content in lysates was determined using Pierce BCA Protein Assay Kit (Pierce Biotechnology, USA). Samples were mass-normalized for electrophoresis via SDS-PAGE in Tris-Glycine buffer using 7.5% gels (cell media) and 5–20% gradient gels (lysates) and electrotransfered onto nitrocellulose membranes. Membranes were stained with Ponceau S and sequentially blocked using 5% non-fat dry milk/1x TBS-T (10 mM Tris-HCl pH 8.0, 150 mM NaCl, 0.5% Tween) for 2 h at RT. Primary antibodies (rabbit anti-collagen-I (1:1000); rabbit anti-osteonectin (1:500); mouse anti-*β*-actin (1:1000) [Novus Biologicals, UK] and rabbit anti-osteocalcin (1:1000) [Biorbyt, UK]) were incubated for 2 h at RT and ON at 4 °C. Secondary antibodies (HRP-linked anti-rabbit and anti-mouse IgGs (1:5000) [GE Healthcare, UK]) were incubated for 2 h at RT and detected by enhanced chemiluminescence (ECL). Protein bands were detected either with ChemiDoc Imaging System (Bio-Rad) and density-analysed on ImageLab (Bio-Rad) or through autoradiography films, scanned using GS-800 Calibrated Densitometer (Bio-Rad) and quantified with Quantity One (Bio-Rad). Quantification of protein bands was analysed as fold change over control levels (non-stimulated).

### Immunocytochemistry and laser scanning confocal microscopy

Briefly, cells grown in coverslips were fixed with 4% paraformaldehyde/PBS (20 min) and permeabilized with 0.2% Triton X-100/1x PBS (10 min). Coverslips were washed (0.1% Tween/1x PBS), blocked for 30 min and incubated with anti-collagen-I antibody (1:250 in 3% BSA/PBST), a FITC-conjugated anti-rabbit secondary antibody (1:300 in 3% BSA/PBST) and a red fluorescing Alexa 568-labelled Phalloidin (1:500). Glass slides were mounted with DAPI-containing Vectashield antifading reagent (Vector, Burlingame, USA) and images were acquired in a LSM 510 META confocal microscope (Zeiss, Germany), as before^48^, in the LiM facility of iBiMED, a node of PPBI (Portuguese Platform of BioImaging).

### Alizarin Red S assays

According to Gregory and Grady Gunn^49^ protocol, after 14 and 28 DIV cells were fixed with 4% paraformaldehyde/PBS and further incubated for 20 min at RT with Alizarin Red S (ARS) (Sigma-Aldrich) stock solution (40 mM, pH 4.1) and images acquired with a camera. To quantify the incorporated stain, 28 DIV cell layers were collected with 10% (v/v) acetic acid, vortexed, heated at 85 °C, incubated on ice, and centrifuged for 15 min at 20,000 g. An aliquot (500 *μ*L) of the supernatant was neutralized with 10% ammonium hydroxide (v/v) (200 *μ*L) and plated in triplicate (150 *μ*L) into a 96-well plate, along with ARS standards (diluted from a 4 mM stock solution). Absorbance was read at 405 nm (Infinite 200 PRO).

### Statistical analysis of biological tests

Raw values were compared to control levels, converted to fold increase values and averaged. The standard error of the mean (SEM) was calculated and data presented as mean ± SEM. Statistical significance analyses of data from control versus data from stripped and interdigitated stimulators were conducted by two-tailed student t-test using the GraphPad Prism software (USA). One-way ANOVA followed by post-hoc analysis were performed to compare biological data between groups (control vs stripped vs interdigitated). Mixed design factorial ANOVA was used to compare variability between and within (along various DIV) groups in the metabolic activity assay.

## Supplementary information

Fig. S1
