# Supplementary material for: Capacitive technologies for highly controlled and personalized electrical stimulation by implantable biomedical systems
Source: Sci Rep. 2019 Mar 21;9:5001. doi: 10.1038/s41598-019-41540-3 (PMC6428833; doi:10.1038/s41598-019-41540-3)
Supplement: Supplementary file 1 — Fig. S1 [file 41598_2019_41540_MOESM1_ESM.pdf]

Capacitive technologies for highly controlled and personalized electrical stimulation by implantable biomedical systems

Marco P. Soares dos Santos [a,b,c,*], J. Coutinho [b], Ana Marote [d], Bárbara Sousa [d], A. Ramos [a,b], Jorge A. F. Ferreira [a,b], Rodrigo Bernardo [b], André Rodrigues [b], A. Torres Marques [e,c], Odete A. B. da Cruz e Silva [d], Edward P. Furlani [f], José A. O. Simões [b], Sandra I. Vieira [d]

[a] *Centre for Mechanical Technology & Automation (TEMA), University of Aveiro, Aveiro, Portugal.*

[b] *Department of Mechanical Engineering, University of Aveiro, Aveiro, Portugal.*

[c] *Associated Laboratory for Energy, Transports and Aeronautics (LAETA)*

[d] *Institute of Biomedicine (iBiMED), Department of Medical Sciences, University of Aveiro, Aveiro, Portugal.*

[e] *Mechanical Engineering Department, University of Porto, 4200-465 Porto, Portugal*

[f] *Department of Chemical and Biological Engineering, Department of Electrical Engineering, University at Buffalo, SUNY, Buffalo, NY, US*

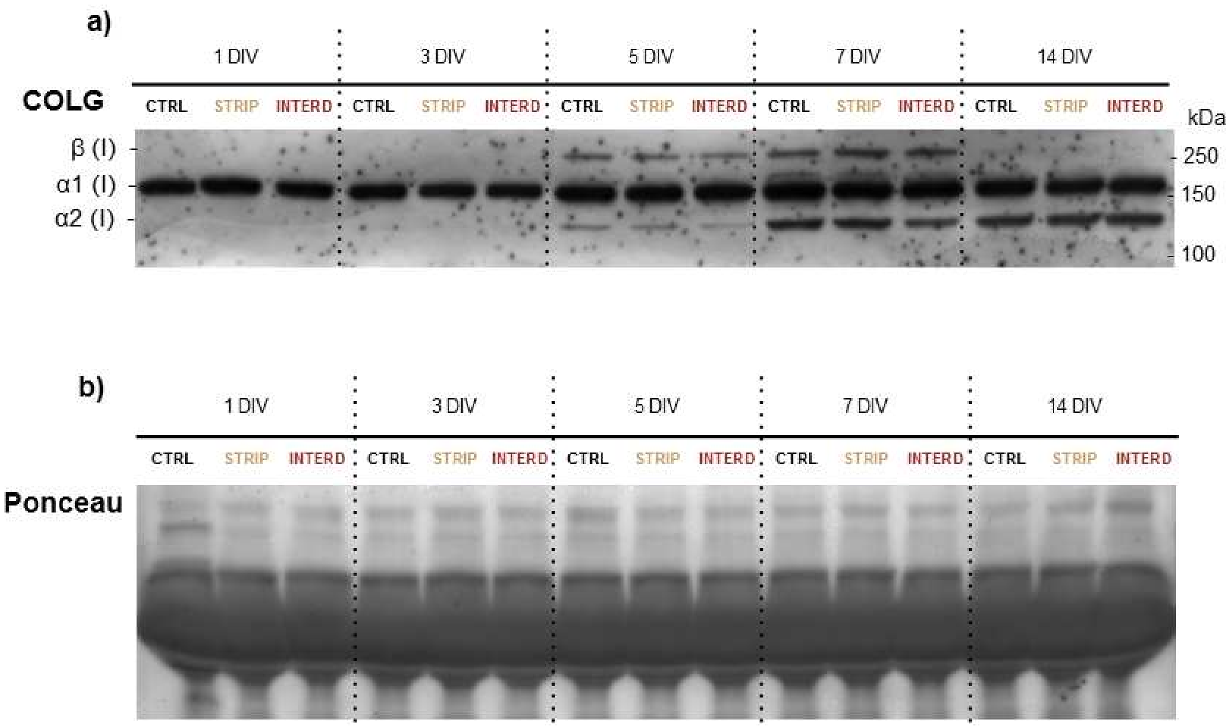

Fig. S1. Immunoblot analyses of time-dependent type-I collagen secretion into the MC3T3 cells' conditioned media in the absence of stimuli (CTRL), and upon low-frequency electric stimulation with two electrode configurations (stripped [STRIP] and interdigitated [INTERD]). a) Immunoblot analysis of unprocessed and processed $\alpha1$(I) and $\alpha2$(I) procollagen monomeric chains (130-160kDa) and β(I), procollagen dimeric forms ($\approx$270kDa). Migration of molecular weight markers is indicated in the right. b) Ponceau S staining of all protein bands present in the blot, used as a loading control for the conditioned media samples. DIV, days *in vitro*. Of note, bands are presented as they are in the gel/blot/film, with no regrouping of cropped parts.

---

* Corresponding author.
Email address: marco.santos@ua.pt (Marco P. Soares dos Santos).
